# Supplementary material for: Inactivation of SARS-CoV-2 virus in saliva using a guanidium based transport medium suitable for RT-PCR diagnostic assays
Source: PLoS One. 2021 Jun 11;16(6):e0252687. doi: 10.1371/journal.pone.0252687 (PMC8195355; doi:10.1371/journal.pone.0252687)
Supplement: Attachment — Submitted filename: PONE-D-21-8255.pdf [file pone.0252687.s001.pdf]

Banik *et. al*. describes the use of a commercially available buffer to inactivate SARS-CoV-2 virus in saliva sample which is compatible with downstream RT-PCR applications. The research question is clear, and the methods used and results are adequate to answer the research question. The paper is well written in general.

Question:
How common is saliva swab/sample being used for SARS-CoV-2 detection compared to nasal swab? Have you tested eNAT inactivation on nasal swab which is commonly used?

Minor comments:
Line 20: spell out RT-PCR

Line 40: instead of “after being opened” it is better to say “during processing”

Line 61: instead of “be used”, it is better to use “help”

Line 68: and 127 Use “SARS-CoV-2” negative instead of “COVID-19”

Line 82, 87, 95, 124, 128, 153, 175 etc.: change “X” to “×”

Line 103: Please add the MWCO for the Slide-A-Lyzer

Line 161, 162: change cell “lines” to “line”

Table 1: Please add the MWCO for “Amicon Ultra 4mL” and Slide A-Lyzer

Table 2 and 3: “Tissue” to “tissue”

Table 2 and 3: I suggest centre the text in the table

Table 2 and 3: “tested by both culture and TCID50” to “tested by both CPE and TCID50”

Line 217: define “LOD”

Statistical analysis
What’s the sample size in Figure 1? If the sample size is small and data is not normally distributed, a non-parametric test (Krusal-Wallis) is more appropriate.

What post hoc test was used on top of ANOVA (or Kruskal-Wallis)? Please provide the information in the manuscript or perform post-hoc test is it hasn’t been done.
